# Shame and Guilt-Proneness in Adolescents: Gene-Environment Interactions

**DOI:** 10.1371/journal.pone.0134716

**Published:** 2015-07-31

**Authors:** Aurora Szentágotai-Tătar, Adina Chiș, Romana Vulturar, Anca Dobrean, Diana Mirela Cândea, Andrei C. Miu

**Affiliations:** 1 Department of Clinical Psychology and Psychotherapy, Babeș-Bolyai University, Cluj-Napoca, Cluj, Romania; 2 Department of Cell and Molecular Biology, Iuliu Hațieganu University of Medicine and Pharmacy, Cluj, Cluj-Napoca, Romania; 3 Cognitive Neuroscience Laboratory, Department of Psychology, Babeș-Bolyai University, Cluj-Napoca, Cluj, Romania; Technion—Israel Institute of Technology, ISRAEL

## Abstract

Rooted in people’s preoccupation with how they are perceived and evaluated, shame and guilt are self-conscious emotions that play adaptive roles in social behavior, but can also contribute to psychopathology when dysregulated. Shame and guilt-proneness develop during childhood and adolescence, and are influenced by genetic and environmental factors that are little known to date. This study investigated the effects of early traumatic events and functional polymorphisms in the brain-derived neurotrophic factor (BDNF) gene and the serotonin transporter gene promoter (5-HTTLPR) on shame and guilt in adolescents. A sample of N = 271 healthy adolescents between 14 and 17 years of age filled in measures of early traumatic events and proneness to shame and guilt, and were genotyped for the BDNF Val66Met and 5-HTTLPR polymorphisms. Results of moderator analyses indicated that trauma intensity was positively associated with guilt-proneness only in carriers of the low-expressing Met allele of BDNF Val66Met. This is the first study that identifies a gene-environment interaction that significantly contributes to guilt proneness in adolescents, with potential implications for developmental psychopathology.

## Introduction

Shame and guilt are negative self-conscious emotions (i.e., involving people’s reactions to their own characteristics and behavior), typically experienced in situations of failure or in which behavioral standards are violated [1,2]. While they are elicited by similar types of situations, shame and guilt differ in terms of how individuals appraise transgressions or errors [2,3], with negative evaluations of the global self in shame, and of specific behaviors in guilt [4].

Research on shame and guilt has mainly focused on childhood [5,6], and relatively little is known about the course and implications of these self-conscious emotions across the life span [7]. Considering that shame and guilt are concerned with how one is being perceived and evaluated by others [8], these emotions may undergo important changes during adolescence [9,10], when emotional reactivity to the social environment increases [11]. Indeed, during adolescence, concern over social evaluation significantly rises compared to childhood [12], self-consciousness peaks [13], and sensitivity to stimuli relevant for self-conscious emotions is higher than in children and adults [14]. Therefore, while shame and guilt-proneness emerge earlier during childhood, they may undergo important changes during adolescence, taking adaptive or maladaptive forms.

### Shame and Guilt-Proneness

Occasional feelings of shame and guilt are functional and serve social goals [1]. For example, shame predicts prosocial behavior and motivation for self-change [15–17]. Likewise, guilt has been associated with higher levels of empathy and prosocial behavior, lower levels of aggression, and lower levels of risky and delinquent behavior in children and adolescents [10,18–20].

However, problems can arise when people's lives are pervaded by shame and guilt, and over the long run, proneness to shame and guilt may play an important role in psychopathology. Studies have consistently linked shame-proneness in children and adolescents with anxiety, depression, eating disorders, externalizing symptoms and delinquent behavior [1,21]. Data regarding the association between guilt-proneness and psychological problems are less consistent [1]. They seem to indicate that guilt over specific behaviors is not associated with poor psychological adjustment [2,22], and that guilt only becomes maladaptive when it is fused with shame, when people develop a distorted sense of responsibility for events beyond their control, and when opportunities for reparation are blocked [2,23,24]. Indeed, similar to shame, maladaptive guilt in children and adolescents correlates with depression [25,26].

Considering the links between shame and guilt-proneness and child and adolescent psychological problems, recent research has focused on factors such as early trauma that may contribute to these emotional dispositions.

### Early Trauma, Shame and Guilt

Early adverse experiences may have lasting effects on people's emotional responses [27,28], including proneness to shame and guilt [29]. Retrospective reports of adults, as well as cross-sectional and prospective studies with children and adolescents indicate that feelings of shame are linked with a history of physical and sexual abuse [30–33], and with various forms of psychological maltreatment such as indifference, rejection, abandonment, neglect, devaluation and shaming by parents [24,34–37]. Moreover, state shame is a mediator between trauma and various outcomes such as depression and post-traumatic stress disorder (PTSD) [30,33,34].

The relation between guilt-proneness and early adverse experiences is less consistent. However, a study by Stuewig and McCloskey [37] found an association between harsh parenting and guilt-proneness in adolescents, while studies by Donatelli and colleagues [38], and Rakow and colleagues [39,40] showed that inappropriate guilt induction by depressed parents predicts maladaptive guilt and internalizing symptoms in children and adolescents.

Early experience may have different emotional outcomes depending on genetic dispositions [41–43]. The only available twin study on maternal reports of shame and guilt suggests that both genetic and environmental factors contribute to proneness to these self-conscious emotions in children between 14 and 24 months of age, with relatively higher contributions of heritability in shame and shared environment in guilt [44]. However, these results relied on a small sample of twins and were not replicated or followed up in gene candidate studies. To our knowledge, no study until now investigated specific gene-environment interactions in shame and guilt proneness.

### The Current Study

The goal of the current study was to explore gene-environment interactions in adolescent shame and guilt-proneness. While the potential influence of early trauma on shame and guilt-proneness has been examined to some extent, candidate genes that may contribute to shame and guilt-proneness by increasing susceptibility to environmental adversities have not been studied until now. However, shame and guilt-proneness have been linked with depression and anxiety [1,21], and therefore, they may involve some common gene-environment interactions.

Although not all studies reported positive or convergent findings, meta-analyses support the view that carrying the low-expressing alleles of the Val66Met polymorphism in the brain-derived neurotrophic factor (BDNF) gene [45] and the 5-HTTLPR polymorphism in the serotonin transporter gene promoter [46,47] is associated with increased depression and anxiety in people with a history of stressful events.

These genetic polymorphisms are biologically plausible candidates for emotional development and psychopathology considering their involvement in neural plasticity [48,49] and stress reactivity [50,51]. BDNF Val66Met affects activity-dependent neural release of BDNF and hippocampal activity [48,49], and a recent study [52] suggests that this polymorphism may increase susceptibility to depression in people with a history of early trauma through neural mechanisms involving reduced gray matter in the hippocampus. Work on 5-HTTLPR mostly focused on amygdala-related neural mechanisms [53–55]. The low-expressing S allele of 5-HTTLPR is associated with reduced gray matter in amygdala [55], and impaired functional coupling in an amygdala-prefrontal circuit related to emotion regulation [53,55]. A study on adolescents [56] found that functional coupling between amygdala and the ventromedial prefrontal cortex is also involved in emotional vulnerability to early trauma associated with the low-expressing allele of 5-HTTLPR. Therefore, BNDF Val66Met and 5-HTTLPR, as well as their interaction with early trauma, may involve different pathways to depression and anxiety, related to the structure and function of hippocampal and amygdala-prefrontal circuits, respectively.

Recent studies have also approached some of the psychological mechanisms that may be involved in the influence of BDNF Val66Met and 5-HTTLPR on emotional dispositions. It was found that children carrying the BDNF Met allele, whose mothers suffered from depression [57], as well as children carrying the 5-HTTLPR S allele [57,58] display a memory bias for negative self-descriptive traits. Preliminary evidence also suggests that 5-HTTLPR may be associated with a tendency to make internal, global and stable attributions for negative events [59]. Considering that these cognitive biases affecting memory and appraisal may contribute to the emergence of emotion dispositions [60], and that they have been linked to shame and guilt [61], this study investigated whether BDNF Val66Met and 5-HTTLPR moderated the relation between early trauma and shame and guilt proneness in adolescents. Based on previous literature, we expected that shame and guilt-proneness are increased in adolescents with a history of early trauma who also carry the low-expressing alleles of BNDF Val66Met and 5-HTTLPR polymorphisms.

## Materials and Methods

### Participants

This study is based on a sample of N = 271 adolescents (165 girls) aged between 14 and 17 years (M = 15.82, SD = 1.169). They were all Caucasians and Romanian was their first language. None of the participants reported diagnosed neuropsychiatric disorders or use of psychoactive medication. Before the study, the procedure was explained to participants and their parents, and participants' assent and written parental consent were obtained. The study followed the recommendations of the Declaration of Helsinki regarding participant safety [62] and was approved by the Ethics Committee of Babeș-Bolyai University.

### Genotyping

DNA was extracted from buccal epithelial cells using the MasterPure Complete DNA & RNA Purification Kit (Epicentre, Madison, USA) and kept at -20°C.

BDNF Val66Met (rs6265) genotyping was performed using tetra primer amplified refractory mutation system (ARMS)-PCR method, as previously described [63]. This polymorphism creates two alleles, Met and Val, the former of which is relatively low-expressing [48]. Considering that the low-expressing Met allele was relatively rare in this sample (frequency: 0.22), which is typical of European samples [64], we aimed to compare Val homozygotes (i.e., Val/Val) and Met carriers (i.e., Val/Met, Met/Met).

Using a previously described protocol [65,66], 5-HTTLPR genotyping included both the insertion/deletion polymorphism, which creates a short (S) allele and a long (L) allele [67], and the neighboring single-nucleotide polymorphism rs25531 that further differentiates the L allele into L_A_ and L_G_ [66]. Based on these related polymorphisms, 5-HTTLPR is considered triallelic, with two low-expressing alleles, S and L_G_ (coded S'), compared to the L_A_ allele (coded L') [68]. The triallelic 5-HTTLPR genotypes were therefore coded as S'S' (i.e., SS, L_G_L_G_ and SL_G_), S'L' (i.e., SL_A_, L_G_L_A_) and L'L' (L_A_L_A_).

### Self-Report Measures

*Test of Self-Conscious Affect for Adolescents* (TOSCA-A) [69]. Shame-proneness and guilt-proneness were assessed using the corresponding scales from TOSCA-A, a dispositional measure of self-conscious emotions. The TOSCA-A scales consist of 15 scenarios, 10 negative and 5 positive, yielding indices of proneness to shame and guilt. Each scenario (e.g., *You and your friend are talking in class*, *and you get in trouble*) is followed by a list of possible responses (e.g., *You would think*: *'I should know better*. *I deserve to get in trouble*'). Participants rate the likelihood of each response on a scale ranging from 1 (not at all likely) to 5 (very likely). Similar to reliability coefficients reported in previous studies [70], Chronbach's alpha in this sample was 0.807 for the shame subscale and 0.854 for the guilt subscale. There was a significant, but moderate correlation (r = 0.409, p < 0.001) between shame and guilt scores in this sample.

*Childhood Traumatic Events Scale* (CTES) [71] is a self-report measure used to identify several types of traumatic events experienced before the age of 17 (or until present in participants of younger ages): (1) physical abuse, such as mugging or assault; (2) sexual abuse, such as rape or molestation; (3) major parental conflicts, divorce or separation; (4) death of a family member or close friend; and (5) severe illness or injury. An additional item refers to other, not specified adverse events. Participants report whether they experienced each type of stressful event and if they did, they are asked to rate its severity on a scale ranging from 1 (not at all) to 7 (extremely). Considering that there is a single item on the occurrence of each stressful event and its severity, it was not possible to analyze the reliability of this scale. However, this scale was used in numerous studies and has shown sensitivity to a wide spectrum of emotional symptoms following early trauma [72,73].

#### Depression and anxiety symptoms

The depression and anxiety scales from the Depression Anxiety Stress Scales [74] were used to assess depression symptoms (e.g., hopelessness, lack of interest) and anxiety symptoms (e.g., subjective apprehension, autonomic arousal). Each of these scales includes 7 items, which are appropriate for adolescents [75] and show good sensitivity to clinical levels of emotional symptoms [76]. Depression scores over 20 and anxiety scores over 14 are considered indicative of clinical problems [74]. In this sample, Cronbach's alpha was 0.710 for the depression scale, and 0.663 for the anxiety scale.

### Statistical Analysis

In order to determine whether genotype frequencies in our sample differed from the general population, departures from the Hardy-Weinberg equilibrium were examined [77]. Skewness was investigated using the Shapiro-Wilk test [78] and significantly skewed variables were normalized using the Box-Cox transformation [79]. Sex differences in shame and guilt-proneness were examined using independent-samples Student t tests, and effect sizes were indicated by Cohen’s d [80].

Gene-environment interactions were analyzed by multiple regressions [81], using the PROCESS macro for SPSS [82].The two genotypes (i.e., BDNF Val66Met, 5-HTTLPR) were separately tested as moderators in the relations between mean intensity of traumatic events (CTES) as predictor, and either shame or guilt-proneness (TOSCA) as outcomes. One dummy code was created for BDNF Val66Met, contrasting Met carriers (coded with 1) and Val homozygotes (coded with 0), and two dummy codes were created for 5-HTTLPR, contrasting either S'S' (coded with 1) with the other genotypes (coded with 0), or S'L' (coded with 1) with the other genotypes (coded with 0) [83]. Slope analysis was used to describe the relations between trauma intensity and shame/guilt-proneness for each genotype [81,83]. Effect sizes for significant moderator effects were indicated by Cohen’s f^2^, indicating the ratio of systematic variance accounted for by the moderator relative to unexplained variance in the criterion [84]. While a priori estimations of statistical power were not performed, post-hoc power estimations for moderator effects were run using the G*Power software [85].

We report significant effects at the traditional level of significance (p < 0.05). However, considering that the effects of the two genotypes were investigated in three regression models, which may have inflated the type I error, the survival of significant effects at a Bonferroni-corrected (p/3) level of significance (p < 0.017) is also reported.

## Results

### Genotypes

BDNF Val66Met genotyping indicated that there were 171 participants with Val/Val genotype, 82 with Val/Met genotype and 18 with Met/Met genotype. These genotypes were in Hardy-Weinberg equilibrium (χ^2^ = 3.380, p > 0.05). Due to the low number of Met homozygotes, they were included together with heterozygotes in a Met carrier group and compared to Val homozygotes.

Triallelic 5-HTTLPR genotyping indicated that there were 65 participants with L'L' genotype, 110 with S'L' genotype, and 68 with S'S' genotype. Due to insufficient DNA, 5-HTTLPR genotyping could not be performed for 28 participants. Considering that the number of girls and boys excluded was similar (15 girls, 13 boys), the sex ratio in the remaining sample was not affected. In addition, shame and guilt proneness and trauma intensity were not significantly different in excluded participants compared to the rest of the sample (all p’s > 0.175). The distribution of 5-HTTLPR genotypes in this sample was in Hardy-Weinberg equilibrium (χ^2^ = 2.171, p > 0.05).

### Early Trauma

Most participants (62%) reported at least one traumatic event. Death of family or close friend was the most frequent trauma (39.48%), followed by severe illness or injury (22.87%), other traumatic event not specified (22.14%), major parental conflicts or separation (18.08%), physical abuse (8.11%) and sexual abuse (1.47%). Death of family or close friend had the highest traumatic intensity (M = 4.046, SD = 1.723), followed by other traumatic events not specified (M = 3.466, SD = 1.534), major parental conflicts or separation (M = 3.061, SD = 1.795), sexual abuse (M = 3.000, SD = 2.708), physical abuse (M = 2.863, SD = 1.780) and severe illness or injury (M = 2.354, SD = 1.146).

### Genes and Environment

BDNF Val66Met and 5-HTTLPR (dummy coded) were separately tested as potential moderators in the relations between mean trauma intensity as predictor and shame and guilt-proneness as outcomes. Considering that girls reported higher levels of shame-proneness (t[269] = 3.504, p = 0.001, d = 0.435) and guilt-proneness (t[181.379] = 5.876, p < 0.001, d = 0.752) compared to boys, sex was included as covariate in all analyses.

Guilt-proneness (Shapiro-Wilk’s W[266] = 0.961, p < 0.001), but not shame-proneness was significantly skewed. Normalized (i.e., Box-Cox transformed) scores were therefore used in all analyses on the former outcome.

None of the participants reported depression symptoms (M = 4.780, SD = 4.143) and anxiety symptoms (M = 5.631, SD = 3.822) over the clinical cut-offs, and these variables were therefore not used as covariates in the moderation analyses. However, shame-proneness significantly correlated with both depression (r = 0.308, p < 0.001) and anxiety (r = 0.254, p < 0.001), whereas guilt-proneness only correlated significantly with anxiety (r = 0.169, p = 0.006).

**Fig 1:**
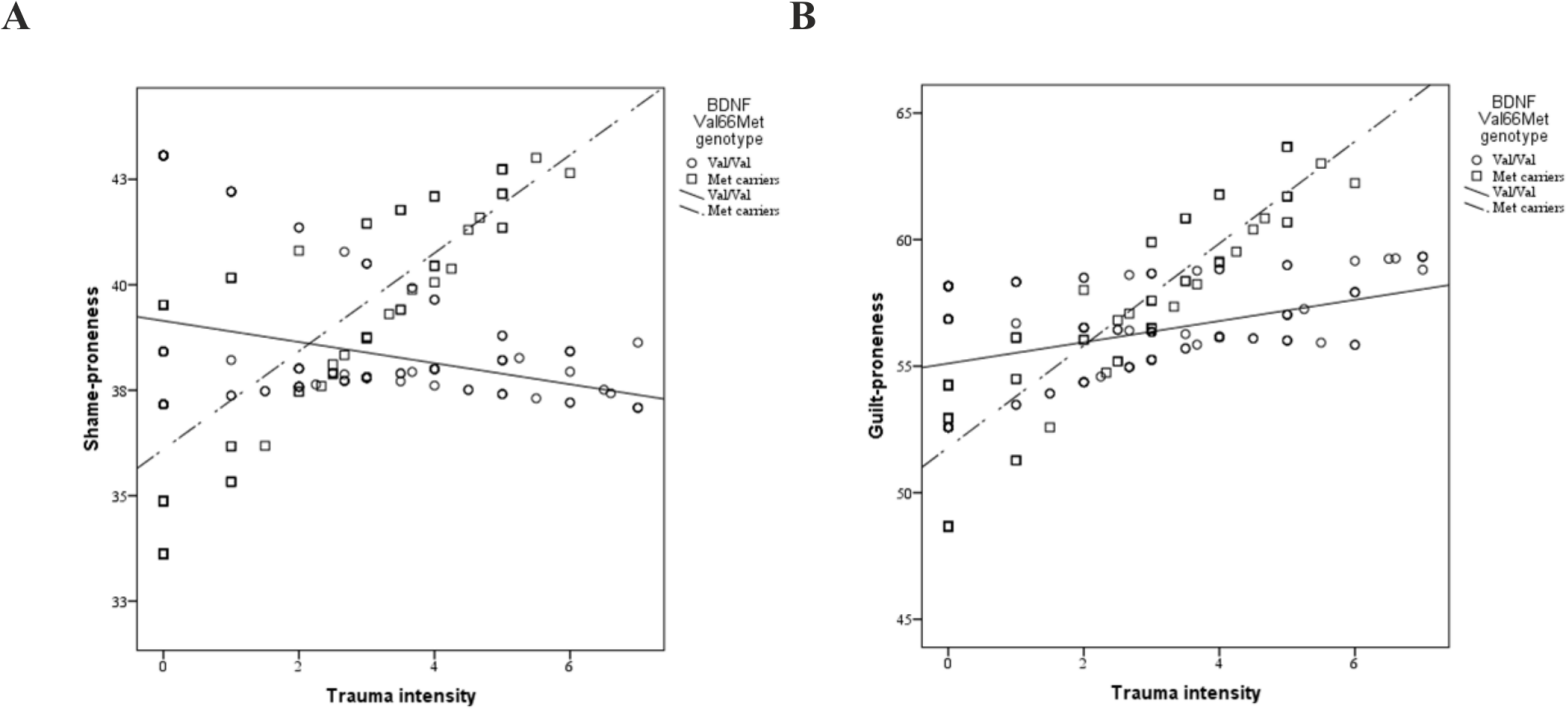
Plot of significant BDNF Val66Met and trauma intensity interaction on shame-proneness (A) and guilt-proneness (B). The slopes describe the relations between trauma intensity and emotional dispositions as a function of genotype.

Results of moderator analyses with shame-proneness as outcome (Table 1) indicated that the main effects of BDNF Val66Met and trauma intensity on shame-proneness were not significant, but their interaction was significant (model 1 in Table 1). Slope analysis indicated that the positive relation between trauma intensity and shame-proneness was significant in BDNF Met carriers (B = 0.981, p = 0.045), but not Val homozygotes (B = -0.373, p = 0.244) (Fig 1). However, this effect did not survive at the Bonferroni-corrected level of significance. In addition, there was a main effect of 5-HTTLPR on shame-proneness when S'S' was contrasted with the other genotypes (model 2 in Table 1), but not when S'L' was contrasted with the other genotypes (model 3 in Table 1). This main effect did not survive at the Bonferroni-corrected level of significance. The interactions between trauma intensity and 5-HTTLPR on shame-proneness were not significant.

**Table 1:** Coefficients for regressing self-reported shame on mean intensity of traumatic events, BDNF Val66Met genotype and 5-HTTLPR genotype, while controlling for sex.

		B	SE B	95% CI
Model 1	BDNF Val66Met dummy (Met carriers coded 1, Val homozygotes coded 0)	-0.117	1.169	-2.418, 2.185
	Mean intensity of traumatic events (centered)	-0.373	0.320	-1.003, 0.256
	BDNF Val66Met dummy × Mean intensity of traumatic events	1.354*	0.582	0.208, 2.501
Model 2	5-HTTLPR dummy 1 (S'S' coded 1, all other coded 0)	2.754*	1.307	0.180, 5.327
	Mean intensity of traumatic events (centered)	0.232	0.338	-0.434, 0.899
	5-HTTLPR dummy 1 × Mean intensity of traumatic events	-0.581	0.602	-1.767, 0.604
Model 3	5-HTTLPR dummy 2 (S'L' coded 1, all other coded 0)	-1.373	1.190	-3.718, 0.971
	Mean intensity of traumatic events (centered)	-0.245	0.366	-0.966, 0.477
	5-HTTLPR dummy 2 × Mean intensity of traumatic events	0.623	0.580	-0.520, 1.766

*Note*. Abbreviations: B, unstandardized regression coefficient; CI, confidence interval; SE, standard error.

* p< 0.05

Table 2 shows the results of moderator analyses with guilt-proneness as outcome. The main effect of BDNF Val66Met and trauma intensity were not significant (model 1 in Table 2), but their interaction was significant and survived at the Bonferroni-corrected level of significance (f^2^ = 0.022). Slope analysis indicated that trauma intensity was positively related to guilt in BDNF Met carriers (B = 1.731, p < 0.001), but not Val homozygotes (B = 0.184, p = 0.538) (Fig 1). The main effects of 5-HTTLPR and its interactions with trauma intensity were not significant (models 1 and 2 in Table 2). Trauma intensity had a significant main effect in one of the models, when S'S' was contrasted with the other 5-HTTLPR genotypes (model 2 in Table 2), but the effect did not survive at the Bonferroni-corrected level of significance.

**Table 2:** Coefficients for regressing self-reported guilt mean intensity of traumatic events, BDNF Val66Met genotype and 5-HTTLPR genotype, while controlling for sex.

		B	SE B	95% CI
Model 1	BDNF Val66Met dummy (Met carriers coded 1, Val homozygotes coded 0)	-0.101	1.137	-2.339, 2.138
	Mean intensity of traumatic events (centered)	0.184	0.342	-0.489, 0.857
	BDNF Val66Met dummy × Mean intensity of traumatic events	1.547**	0.564	0.436, 2.658
Model 2	5-HTTLPR dummy 1 (S'S' coded 1, all other coded 0)	2.175	1.335	-0.455, 4.806
	Mean intensity of traumatic events (centered)	0.889*	0.347	0.205, 1.572
	5-HTTLPR dummy 1 × Mean intensity of traumatic events	-0.211	0.613	-1.419, 0.997
Model 3	5-HTTLPR dummy 2 (S'L' coded 1, all other coded 0)	-1.956	1.171	-4.263, 0.350
	Mean intensity of traumatic events (centered)	0.720	0.373	-0.014, 1.455
	5-HTTLPR dummy 2 × Mean intensity of traumatic events	0.199	0.598	-0.979, 1.377

*Note*. Guilt-proneness scores were normalized using the Box-Cox transformation. Abbreviations: B, unstandardized regression coefficient; CI, confidence interval; SE, standard error.

* p < 0.05

** p < 0.01

### Supplementary Analyses

We investigated shame and guilt-proneness in relation to each type of traumatic event. Participants who reported experiences of severe illness or injury had increased shame-proneness compared to those who did not have such experiences (t[264] = 2.204, p = 0.028, Cohen's d = 0.317). There were no other significant differences between participants who reported other types of traumatic events (i.e., physical abuse; sexual abuse; major parental conflicts or separation; death of family of close friend) and those who did not.

## Discussion

The main finding of this study is that the intensity of early trauma in adolescents is positively related to guilt-proneness only in carriers of the low-expressing Met allele of BDNF Val66Met. Other positive relations between the 5-HTTLPR S'S' genotype and shame-proneness, and trauma intensity and guilt proneness, as well as the interaction between BDNF Val66Met and early trauma on shame-proneness were also found, but these effects did not survive at the level of significance corrected for multiple testing and are thus less reliable.

This is the first gene-environment interaction study on shame and guilt-proneness, but it adds to a recent line of research that has linked early trauma and BDNF Val66Met to negative self-evaluation. For example, maternal depression has been associated with a memory bias toward negative self-descriptive traits in children carrying the Met allele of BDNF Val66Met [57]. Using a similar approach, another study found that adult men with a history of early trauma and the BDNF Met allele show reduced memory for positive self-referential descriptors [86]. These results, along with the present findings, suggest that the low-expressing allele of BDNF Val66Met may increase emotional vulnerability following early trauma through biased negative self-evaluation and related emotional dispositions such as guilt-proneness. Guilt-proneness in adolescents may be an important facet of this "depressogenic" phenotype, considering its prospective association with depression [37]. In this study, which focused on healthy adolescents, guilt-proneness correlated with subclinical anxiety symptoms, which suggests that higher levels of guilt are maladaptive. Shame-proneness was also positively associated with subclinical depression and anxiety symptoms, and like guilt-proneness, this emotional disposition was affected by the BDNF Val66Met and early trauma interaction. However, this effect did not survive at the corrected level of significance and should be interpreted with caution until more convincing evidence emerges. Post-hoc power analyses based on effect sizes and sample characteristics indicated that power to detect a gene-environment interaction on shame-proneness was suboptimal (0.515) and lower than for guilt-proneness (0.995) in this study, which suggests that future attempts to replicate potential effects on the former outcome should be based on larger samples.

The present finding raises the question of the neural mechanisms by which the BDNF Val66Met and early trauma interaction influences guilt-proneness in adolescents. Recent neuroimaging work suggested that the hippocampus plays a central role in the neural pathway that links BDNF Val66Met and emotional vulnerability following early trauma. A history of early trauma in adults carrying the BDNF Met allele is associated with reduced hippocampal gray matter and depression [52]. In addition, a functional study found increased hippocampal activity during processing of emotional facial expressions in adolescents suffering from depression who also carried the BDNF Met allele [87]. Other neural structures such as the insula may be involved in the link between maladaptive guilt and depression [88], but their anatomical and functional modulation by BDNF Val66Met and early trauma has not been studied. To date, neuroimaging genetics research shows that the BDNF Val66Met and early trauma interaction affects brain structure and function, and its influence on the hippocampus may be particularly relevant for long-term emotional vulnerability.

Trauma intensity in adolescents was not related to shame-proneness, but was positively related to guilt-proneness. However, this effect was significant in only one of the models (i.e., 5-HTTLPR, with S'S' vs. all others × trauma intensity) and did not survive at the corrected level of significance. This is in line with recent evidence suggesting that childhood trauma influences shame and guilt-proneness only indirectly. In a study conducted by Stuewig and McCloskey [37], the authors investigated the longitudinal paths between child maltreatment, parenting and shame and guilt-proneness during adolescence. They found that sexual abuse and parental conflict, two forms of early trauma also investigated in this study, were not significantly associated with shame and guilt-proneness, but that harsh parenting (i.e., punitive practices of yelling, hitting, and spanking) during childhood contributed to shame and guilt-proneness in adolescence, through later parental rejection (i.e., excessive criticism and humiliation) [37]. Therefore, early trauma may not have direct effects on proneness to shame and guilt, which is not surprising considering that it is temporally distal relative to emotional dispositions in adolescence. An earlier cross-sectional study in adults also found that emotional, but not physical abuse was related to shame-proneness, and neither was related to guilt-proneness [89]. In apparent contrast, there are studies reporting that shame is associated with sexual and physical abuse, and plays a mediator role in the relation between abuse and depression or PTSD [30–33]. However, these studies did not assess shame-proneness, but domain-specific feelings, such as bodily shame [30], or event-specific feelings, such as shame about being the victim of a violent crime [31] or sexual abuse [32,33]. In this study, we found that only adolescents with a history of severe illness or injury have increased shame-proneness. Overall, while it is possible that the limited effects of early trauma intensity on shame and guilt-proneness may be related to the small cell sizes of certain abuse groups (e.g., sexual abuse, physical abuse) in this study, these results are in line with the two other studies that also assessed dispositional shame and guilt [37,89].

There was only one main effect of 5-HTTLPR, with higher shame-proneness in adolescents with the S'S' genotype. However, this effect did not survive the correction for multiple testing. Failing to find an interaction between 5-HTTLPR and early trauma in relation to shame and guilt may seem surprising considering the literature supporting the effect of this interaction on depression and anxiety [46]. However, a recent meta-analysis [47] emphasized that most studies on 5-HTTLPR, stress and depression are focused on adults between 21 and 50 years of age, and life stressors rather than childhood stressors and illnesses. Therefore, differences in age of participants and type of trauma may explain the apparent divergence from the depression literature. In addition, this study took a triallelic approach to 5-HTTLPR genotyping, which has only recently started to be employed in gene candidate studies [66]. By differentiating two functionally distinct L alleles, genotyping rs25531 contributes to more accurate identification of 5-HTTLPR genotypes and reduces the likelihood of false positive findings [65,66]. The lack of evidence for a significant 5-HTTLPR and early trauma interaction may suggest that the neural mechanisms (e.g., amygdala-prefrontal circuits) underlying its influence on depression and anxiety [56] may differ from those involved in gene-environment interactions influencing shame and guilt-proneness.

The implications of these results are related to the possibility of targeting shame and guilt-proneness in prevention programs aiming to reduce the risk of psychopathology in adolescents [37]. Based on this study, adolescents with higher levels of childhood trauma and the BDNF low-expressing allele are primary candidates for prevention programs focused on anxiety and depression and other forms of psychopathology related to increased guilt. Recent studies in therapygenetics [90] have also started to examine the influence of BDNF Val66Met on psychotherapy outcomes [91,92] and in the future, they might uncover clinical aspects of the interaction between BDNF Val66Met and early trauma. However, one must consider that the effect size of this interaction is small [80] in this study, which is not surprising considering that like most psychological characteristics [93,94], shame and guilt-proneness are complex or polygenic phenotypes that probably involve hundreds or thousands of gene polymorphisms and their interactions with environment. With an increasing interest in shame and guilt-proneness as potential markers of risk for developmental psychopathology [1,21], future studies might take the challenge of uncovering other relevant gene-environment interactions.

This study has at least three limits. First, the sample is small in light of estimated sizes that would be needed in gene candidate studies focused on functional polymorphisms with small effects [95]. Therefore, these results should be taken with caution until they are independently replicated. Second, three-way interactions between BDNF Val66Met, 5-HTTLPR and early trauma could not be tested in this study because of limited statistical power. While the interaction between BDNF and the serotonin transporter molecule is biologically plausible [96] and relevant for emotional disorders [97], larger sample sizes are needed in order to examine gene × gene and gene × gene × environment interactions. A recent study [98] indicates that the moderator effects of BDNF Val66Met and 5-HTTLPR in the relation between childhood adversity and depression are independent. This suggests that not including three-way interactions in statistical models does not affect the potential of uncovering gene-environment interactions separately implicating the two polymorphisms. Finally, the recency of trauma was not assessed in this study. The questionnaire that we have used is typical for the early life stress literature and has been previously employed in adolescent studies [73], but it did not allow us to control for developmental timing of trauma [99]. However, recent studies that have differentiated between childhood adversity and recent stress found that only the former interacts with BDNF Val66Met in relation to emotional responses [100,101], which suggests that this study may have tapped the effect of early trauma.

In conclusion, our results indicate that BDNF Val66Met is a significant moderator of the relation between early trauma and guilt-proneness in healthy adolescents. Should these results be replicated in independent samples, future studies might focus on the neural mechanisms underlying the effect of this gene-environment interaction on proneness to negative self-conscious emotions and their impact on developmental psychopathology.
